# Thermo-sensitive composite hydrogels based on poloxamer 407 and alginate and their therapeutic effect in embolization in rabbit VX2 liver tumors

**DOI:** 10.18632/oncotarget.11789

**Published:** 2016-09-01

**Authors:** Lili Huang, Ming Shen, Rongxin Li, Xiangyu Zhang, Ying Sun, Pei Gao, Hao Fu, Hongqiang Liu, Yang He, Yuqing Du, Jun Cao, Yourong Duan

**Affiliations:** ^1^ State Key Laboratory of Oncogenes and Related Genes, Shanghai Cancer Institute, Renji Hospital, Shanghai Jiao Tong University School of Medicine, Shanghai, China; ^2^ Department of Interventional Oncology, Dahua Hospital, Xuhui District, Shanghai, China; ^3^ Department of Interventional Radiology, Shanghai Pudong New District Punan Hospital, Shanghai, China

**Keywords:** thermo-sensitive composite hydrogels, poloxamer 407, sodium alginate, sol-gel transition, transarterial embolization

## Abstract

Interventional embolization therapy is an effective, most widely used method for inoperable liver tumors. Blood-vessel-embolic agents were essential in transarterial embolization (TAE). In this work, thermo-sensitive composite hydrogels based on poloxamer 407, sodium alginate, hydroxymethyl cellulose and iodixanol (PSHI), together with Ca2+ (PSHI-Ca2+) were prepared as liquid embolic agents for TAE therapy to liver cancer. With increasing temperature, PSHI exhibited two phase states: a flowing sol and a shrunken gel. Rheology tests showed good fluidity and excellent viscoelastic behavior with a gelation temperature (GT) of 26.5°C. The studies of erosion indicated that PSHI had calcium ion-related erosion characteristics and showed a slow erosion rate in an aqueous environment. When incubated with L929 cells, the thermo-sensitive composite hydrogels had low cytotoxicity *in vitro*. The results of analyzing the digital subtraction angiography and computed tomography images obtained from *in vitro* and *in vivo* assays indicated a good embolic effect in the renal arteries of normal rabbits. Angiography and histological studies on VX2 tumor-bearing rabbits indicated that PSHI-Ca2+ successfully occluded the tumors, including the peripheral vessels. In conclusion, PSHI-Ca2+ was a promising embolic agent for transarterial embolization therapy.

## INTRODUCTION

Hepatocellular carcinoma (HCC) is one of the most common cancers worldwide and the third most common cause of cancer-related deaths [1]. Recently, the development of minimally invasive procedures including radiofrequency ablation (RFA) [2], percutaneous ethanol injection (PEI) [3], transarterial embolization (TAE) [4] and transarterial chemoembolization (TACE) [5] have played increasingly important roles in unresectable liver tumors. Interventional embolization therapy is an effective, first-line method for inoperable liver tumors and plays an important role in various diseases, including AVMs [6], hemorrhages [7], uterine fibroids [8] and lung cancer [9].

Industrial advances have led to the clinical application of many materials for the occlusion of blood vessels. Granular embolic particles such as polyvinyl alcohol (PVA) are a type of permanent embolic agent due to their high effectiveness for interventional therapy. Due to their large size, mircoparticles were difficult to pass through a mircocatheter and enter the peripheral arteries [10, 11]. Cyanoacrylates and Onyx have been widely used for many years as clipping and effective liquid embolic agents [12]. However, these materials must be dissolved in organic solvents such as dimethyl sulfoxide, which is toxic to normal tissues [13]. Therefore, a successful embolic agent for embolization therapy should meet the requirements of entering the peripheral blood vessels, where it develops high strength and provides drug loading abilities, especially in liver cancer.

Thermo-sensitive hydrogels are polymer materials that respond to small temperature changes to form a physically cross-linked hydrogel via a sol-gel phase transition. Hydrogels are easy to inject because they are free-flowing liquids with a lower critical solution and are transformed into a gel at body temperature. Some studies have also found that copolymers such as injectable hydrogels can be used as subcutaneous implants that are applied via minimally invasive procedures [14]. These copolymers are three-dimensional hydrophilic polymer networks that contain considerable water, which is nontoxic, and have rapidly reversible sol-gel transition behavior with changes in temperature [15]. Raymond studied poloxamer 407 as a temporary temperature-sensitive embolic agent in the arteries of several animals because of its fast erosion [16]. Adding macromolecules to a poloxamer gel has been proposed for the construction of composite hydrogels to improve their sustained release properties [17]. The copolymers can reinforce the poloxamer network by scaffolding or the interaction between the micelles and the network. Previous reports have shown that adding carrageenan, dextran and chitosan macromolecules to poloxamer 407 decreased its *in vitro* erosion and increased the intravaginal residence time [18, 19]. In addition, studies reported calcium ion cloud formed hydrogels with alginate via intramolecular bridges [20]. For these reasons, we added alginate into poloxamer 407 to increase the residence by calcium ions. In this study, we prepared a thermo-sensitive embolization delivery system based on sodium alginate and hydroxymethyl cellulose (HPMC) loaded with poloxamer 407 for the treatment of liver cancer. These composite hydrogels should be injectable at ambient temperature (<25°C) and transformed into a gel at body temperature (37°C). To optimize and demonstrate the potential of the composite hydrogels, we investigated the *in vitro* effect of the iodixanol and poloxamer 407 concentrations on the hydrogel behavior, rheology and erosion. Furthermore, the *in vivo* potential embolic efficiency of the thermo-sensitive composite hydrogels was studied in the renal arteries of normal rabbits and in VX2 tumors by angiography, computed tomography, and histological methods.

## RESULTS

### Sol-Gel transition of a PSHI aqueous solution

**Figure 1:**
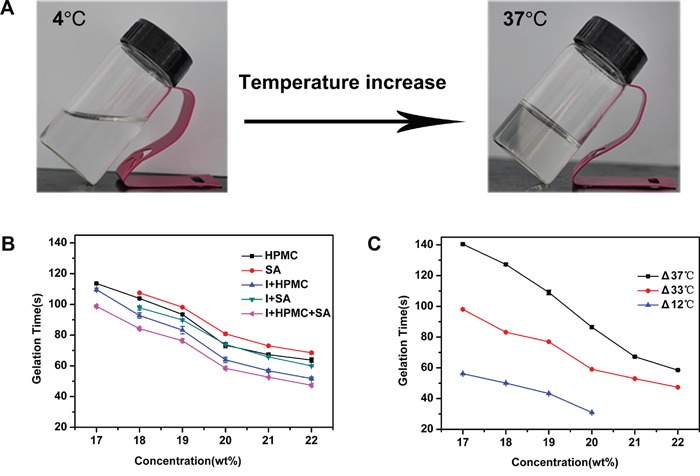
The Sol-Gel transition characteristics of a thermo-sensitive gel **A.** Phase changes with a temperature increase. **B.** The gelation time of different formulations with increasing concentrations of P407. “SA” indicates alginate, “I+SA” indicates alginate and iodixanol, “HPMC” indicates hydroxymethyl cellulose, “I+HPMC” indicates iodixanol and hydroxymethyl cellulose, “I+HPMC+SA” indicates iodixanol, hydroxymethyl cellulose and alignate. **C.** The gelation time of a thermo-sensitive gel influenced by different temperature differences (ΔT).

A solution with a high poloxamer 407 (P407) concentration (17%-22% w/v) was clear at 4°C. Figure 1A shows an example of a thermo-sensitive composite hydrogel set in an inverted tube. When the composite copolymer concentration reached the critical gelation concentration, the PSHI assumed transitioned from an aqueous solution to a gel as the temperature was increased from 4°C to 37°C. Solutions containing 0.5% sodium alginate (SA) had a shorter gelation time as the P407 concentration was increased from 18% w/v (gelation time = 110 s) to 22% w/v (gelation time = 80 s) (Figure 1B). When HPMC was added to the P407 solution for increasing the tightness of three-dimensional hydrophilic polymer networks, the same phenomenon occurred [15]. For visualization, a non-toxic x-ray contrast agent (Iodixanol) was added to the solution. When the iodixanol (23% v/v) was added, the gelation time decreased (Figure 1B), possibly because the hydrophilic iodixanol led to an increase in the overall hydrophilicity of the system. However, the incorporation of the iodixanol into a P407/alginate/HPMC solution did not change the trend of the gelation time curve (Figure 1B).

The difference between the temperature of the PSHI before injection and the body temperature changed the sol–gel transition, as shown in Figure 1C. The gelation time of the thermo-sensitive composite hydrogel decreased as the temperature difference (ΔT) decreased from 37°C to 12°C (Figure 1C). These results allowed the sol-gel transition phase characteristics to be adjusted by altering the temperature difference and the formulation for TAE therapy with an embolic agent.

### Rheological analysis of PSHI aqueous solution

**Figure 2:**
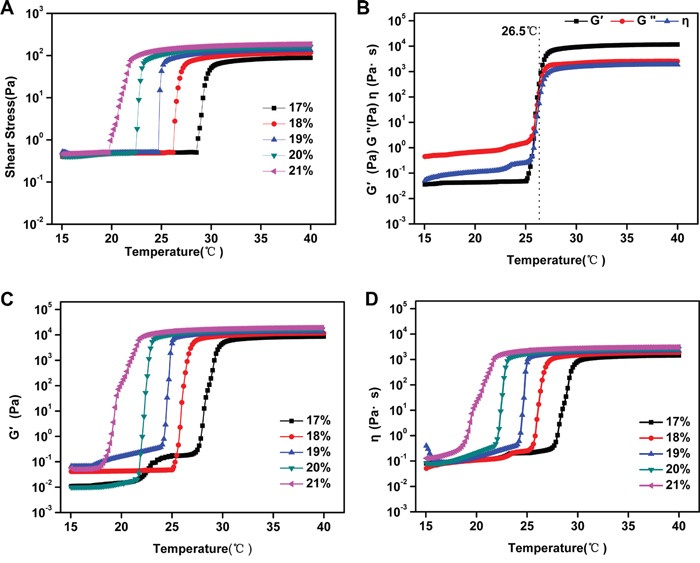
**A.** The shear stress of PSHI with a temperature increase. **B.** The total viscoelasticity of 18% PSHI. The storage modulus (G’) and the viscosity (η) of PSHI **C-D.** with P407 at different concentrations (17%, 18%, 19%, 20% and 21%, respectively) with a temperature increase.

The formation of the thermo-sensitive composite hydrogels was examined using rheometry. The viscosity of PSHI was evaluated at a fixed frequency of 1Hz and a heating rate of 1°C/min. Figure 2A shows the effect of a temperature increase from 15°C-40°C on the shear stress. The shear stress of the PSHI samples increased from 0.5 Pa to 200Pa as the temperature was increased and reached equilibrium at 32°C. The viscosity increased from approximately 0.2 to 3000 Pa s as the temperature was increased (Figure 2B). Figure 2B shows the viscosity data for an 18% P407 PSHI solution with a gelation temperature (GT) of 26.5°C. The storage modulus (G’) and viscosity (η) of the PSHI samples increased with temperature from 15°C to 26.5°C. Above the gelation temperature, the storage modulus (G’) and the viscosity (η) stabilized as the temperature increased. These results demonstrated that the composite hydrogel had a thermo-sensitive response and that the structure of the copolymers was stable. In this study, PSHI solution showed better flowability at room temperature and was suitable for TAE treatment.

The storage modulus (G’), loss modulus (G“) and viscosity (η) measure the interaction effects of the size and structure of a macromolecule in a given solvent [21]. Poloxamer 407 exhibited a thermo-reversible sol-gel transition as the temperature changed [22]. In Figure 2C-2D, G’ and η increased as the P407 concentration increased. At the gelation temperature, G’ and η increased as the temperature increased. Above the gelation temperature, G’ and η stabilized as the temperature increased. These results indicated that P407 was essential to the thermo-sensitive characteristics of PSHI. A liquid embolic agent must have a high strength, but PSHI had a low strength. As previous study showed that a calcium cloud formed a gel in the presence of alginate [23]. The addition of calcium to the poloxamer gel was proposed to improve the strength of the PSHI in further studies.

### Influence of calcium ion on erosion of PSHI

**Figure 3:**
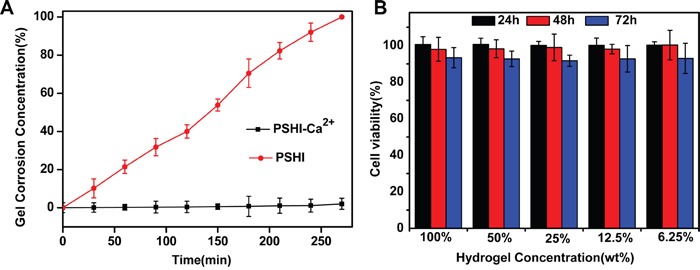
**A.** Erosion of PSHI-Ca2^+^ incubated with PBS compared to PSHI alone. **B.** *In vitro* cytotocity of a PSHI-Ca^2+^ hydrogel in L929 cells. Data are expressed as the Mean±SD.

The erosion behavior of 18% PSHI was investigated. As shown in Figure 3, the presence of calcium ions notably influenced the hydrogel erosion rate of PSHI. Results showed that approximately 50% PSHI dissolved within 150 min when incubated in PBS. When calcium was added, the weight of hydrogel remained unchanged for 5 h. As we tested, the maximum calcium loading capacity was 8.08±0.08 mg/ml PSHI solution. Obviously, PSHI was an interesting hydrogel of which the erosion was controlled by the calcium ion in aqueous environment. This phenomenon might be a result of interactions of the calcium, P407, alginate and HPMC.

### *In vitro* cytotoxicity study

The *in vitro* cytotoxicity of a PSHI-Ca^2+^ hydrogel was investigated via the MTT assay in a mouse fibroblast (L929) cell line. As shown in Figure 3B, the L929 cells remained over 90% viable after they were exposed to extracts of thermo-sensitive hydrogel at different concentrations, demonstrating no obvious cytotoxicity of the PSHI-Ca^2+^ to the L929 cells. The results indicated that the composite hydrogel was no cytotoxic when applied in embolic delivery.

### Renal artery embolization of PSHI-Ca^2+^ in normal rabbits

**Figure 4:**
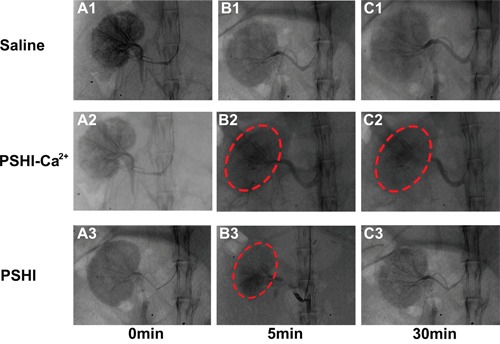
DSA images of normal rabbit kidneys after renal artery embolization with different formulations: Saline (Plot B1), PSHI-Ca^2+^ (Plot B2) and PSHI (Plot B3) Plots A1, A2, and A3 correspond to DSA images before renal artery embolization, respectively. Plots C1, C2, and C3 correspond to DSA images 30 min after renal artery embolization, respectively. The red circles indicate the embolized kidney.

PSHI, as visualized by a contrast agent in the renal arteries, was distributed in the embolized kidneys (Figure 4). A boundary existed between occluded and non-occluded arteries. The black shadow (red arrow) indicates the arteries without an embolism, and the gray shadow shows the occluded arteries in rabbit kidney (Supplementary Figure S1). When 18% PSHI was injected, the small arteries and the peripheral shadow faded; only large arteries were legible in Supplementary Figure S1A. As mentioned previously, an appropriate P407 concentration must be carefully selected for peripheral arterial embolization [24]; therefore, 18% PSHI was chosen.

Further experiments on the embolization efficacy were carried out in the right kidney of rabbits using Saline, PSHI and PSHI-Ca^2+^ as embolic agent. DSA images at various times are shown in Figure 4. Before embolization, the contrast agent iodixanol was injected to fill the blood vessel for superselection. As the DSA images in Figures 4A1-4A3 showed, the blood vessels were clearly evident before embolization. After embolization, the iodixanol solution was injected again to evaluate the embolic efficacy. The solution did not diffuse into the embolized kidney but refluxed to other unembolized blood vessels, as treated by PSHI-Ca^2+^ (Figure 4B2). No obvious difference in the DSA images was found before (Figure 4A1) and after embolization with saline (Figure 4B1-4C1). Five minutes after embolization with PSHI, the renal arteries were gray, which indicated that they were successfully occluded (Figure 4B3). However, 30 min after embolization, the renal arteries were visible again, which suggested that the arteries were recanalized (Figure 4C3). In contrast, the renal arteries remained gray with PSHI-Ca^2+^ after 30 minutes (Figure 4B2-4C2), suggesting that the arteries were successfully embolized. These results demonstrated that PSHI-Ca^2+^ had an excellent embolic efficacy for the peripheral arteries.

**Figure 5:**
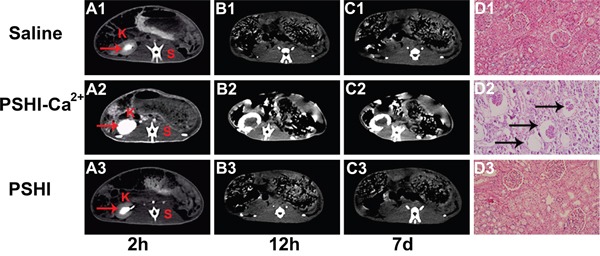
CT scans of normal rabbits at predetermined intervals after embolization using saline (A1-C1), PSHI-Ca^2+^ (A2-C2) and PSHI (A3-C3) K indicates kidney and S indicates spine. Plots D1, Plot D2 and Plot D3 show the kidney histopathology in rabbits 7days after embolization.(original magnification×100)

The embolization efficacy was subsequently assessed by CT. Figure 5 shows CT images at predetermined intervals after embolization with saline (Figure 5A1-5C1), PSHI-Ca^2+^ (Figure 5A2-5C2) and PSHI (Figure 5A3-5C3). After occlusion for 2 h, the medulla of the right kidney became white, clearly because the iodixoanol solution had not completely dissipated, as shown in Figure 5A1. The same phenomenon occurred after a rabbit was treated with PSHI (Figure 5A3), implying that the renal arteries were recanalized. The contour of the kidney was clearly apparent after embolization with PSHI-Ca^2+^ (Figure 5A2), suggesting that the renal arteries had been successfully embolized. The medullary white zone completely disappeared 12 h after embolization with saline (Figure 5B1) and PSHI (Figure 5B3), indicating that the iodixanol was fully dissipated and that the renal arteries were unembolized. The white zone of the kidney cortex is still evident in Figure 5B2 due to the embolic efficacy of PSHI-Ca^2+^. Moreover, 7 days after embolization, the kidney medulla could not be identified in the saline (Figure 5C1) or PSHI (Figure 5C3) treatments. In the PSHI-Ca^2+^ groups, a white ring surrounding the kidney could still be observed (Figure 5C2), indicating that the PSHI-Ca^2+^ had excellent embolic efficacy for 7days. The size and location of the white zone indicate an occluded kidney. These observations further demonstrate a good embolic efficacy of PSHI-Ca^2+^.

H&E staining was used to evaluate the embolic efficacy 7 days after embolization. Figure 5 shows the histology of a kidney embolized with saline (Figure 5D1), PSHI-Ca^2+^ (Figure 5D2), and PSHI (Figure 5D3) after 7 days. No obvious histological changes in the structure and morphology of the renal tissues were noted in the saline (Figure 5D1) and PSHI (Figure 5D3) groups. However, Figure 5D2 shows that the glomeruli of the right kidney were atrophied in PSHI-Ca^2+^ group, indicating successful embolization by PSHI-Ca^2+^ (see the regions indicated by black arrows).

### TAE therapy with composite hydrogels in VX2 liver tumors in rabbits

**Figure 6:**
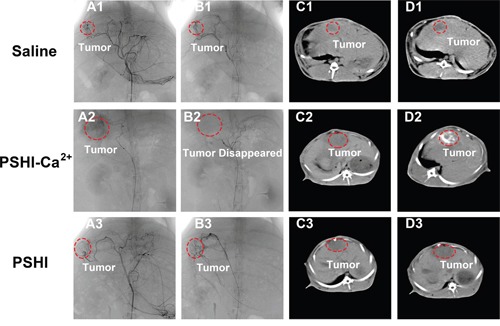
DSA images of VX2 liver tumors in rabbits after embolization with saline (B1), PSHI-Ca^2+^ (B2) and PSHI (B3), A1, A2 and A3 are DSA images before embolization CT images of rabbits bearing VX2 tumors after treatment with saline (D1), PSHI-Ca^2+^ (D2) and PSHI (D3), C1, C2 and C3 are corresponding CT images before embolization.

The TAE treatment with thermo-sensitive composite hydrogels containing PSHI-Ca^2+^ was performed in rabbit VX2 liver cancer model. DSA images at different times are shown in Figure 6. Idoixanol was immediately injected into the tumor vessels before embolization and was used to locate the tumor. No significant differences were evident between pre-embolization and post-embolization with saline (Figure 6A1-6B1). The same phenomenon occurred after treated with PSHI (Figure 6A3-6B3). After treatment with PSHI-Ca^2+^, the black shadow disappeared, which indicated that all of the peripheral blood vessels of the tumor were thoroughly occluded (Figure 6B2). When iodixanol was injected again to test the embolic efficacy, it did not diffuse into the peripheral blood vessels of tumors, but the liver arteries were visualized. A previous study showed that the peripheral occlusion was considerable and inhibited collateral circulation and tumor growth [25]. These results indicate that PSHI-Ca^2+^ successfully embolized and inhibited tumors when it was used as a liquid embolic agent. Furthermore, the embolic efficacy was evaluated by CT and histological analysis. The tumor zone showed no obvious differences in the saline group (Figure 6C1-6D1) and PSHI (Figure 6C3-6D3), but a white zone in the tumor was clearly apparent 4 h after treatment with PSHI-Ca^2+^ (Figure 6D2), suggesting that the tumor was completely occluded.

**Figure 7:**
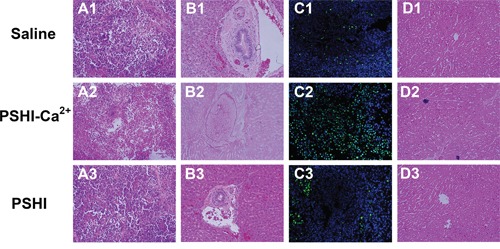
Histopathological H&E staining, TUNEL assay and toluidine blue-eosin staining set of tumor and embolic arterial sections after treatment with saline (A1-D1), PSHI-Ca^2+^ (A2-D2) and PSHI (A3-D3) (original magnification×100).

Histological detection was performed via H&E staining, a TUNEL assay and toluidine blue-eosin staining set. H&E staining was carried out on tumor and arterial sections. A large number of dividing cells with different shapes and various-sized nuclei was observed in the tumor mass. Karyolysis, pyknosis and karyorrhexis were observed in the PSHI-Ca^2+^ group, implying tumor necrosis (Figure 7A2). In the PSHI-Ca^2+^ group (Figure 7B2), the endometrial structure of the embolized hepatic arteries surrounding the tumor was deformed in comparison to the saline (Figure 7B1) and PSHI (Figure 7B3) groups. These results indicated that the hepatic arteries were shriveled and blocked as a result of hypoxia, further demonstrating that the tumor was successfully embolized by PSHI-Ca^2+^. Furthermore, the TUNEL assay (Figure 7C2) showed that the group treated with PSHI-Ca^2+^ was highly fluorescent, indicating a high apoptosis ratio of the tumor cells, which could be a result of hypoxia in the tumor cells. The toluidine blue-eosin staining set (Figure 7D2) illustrated the distribution of blue agglomerates of composite hydrogels in the hepatic arteries. These results further supported a good embolic efficacy of PSHI-Ca^2+^.

### Safety of the composite hydrogel treatment in VX2 liver tumors of rabbits

**Figure 8:**
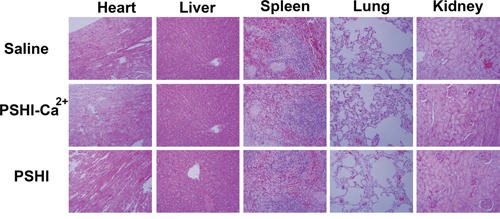
Histological H&E staining of important organs after tumor embolization (original magnification×50).

The systemic toxicity of composite hydrogels was investigated by H&E staining in tissue sections, including heart, liver, spleen, lungs and kidney. At the end of the animal tests, the rabbits were sacrificed, and their organs were sectioned and H&E stained for morphological analysis. As shown in Figure 8, no hemorrhaging, thrombi or abnormalities were found in all organs implying no obvious damage to these organs after each treatment.

## DISCUSSION

In this study, we prepared composite thermo-sensitive PSHI-Ca^2+^ hydrogels as embolic agents for TAE therapy of liver cancer. Previous studies indicated that an appropriate gelation temperature for a thermo-sensitive hydrogel allowed the hydrogel to be in the liquid phase at room temperature and transformed to the gel phase at body temperature [26]. Rheological studies indicated that PSHI had a lower viscosity and exhibited flowable-sol phases as the temperature changed. Viscosity results confirmed that PSHI solutions were suitable for injecting through catheters with a GT of 26.5°C.

Compared with PSHI, PSHI-Ca^2+^ showed a slow erosion rate in an aqueous environment. A possibly mechanism of the effect of calcium on PSHI erosion is displayed in Scheme 1. A calcium ion cloud forms intermolecular bridges via ionic bonds with the carboxyl group in alginate, resulting in the gelification of the alginate [23]. A similar process occurs when a PSHI solution comes into contact with calcium. Before the PSHI comes into contact with the calcium, the alginate exists as a network that is seemingly absorbed on the poloxamer 407 micelles. As the temperature increases, the poloxamer 407 micelles form a close three-dimensional hydrophilic polymer networks, leading to a sol-to-gel phase transition. In the presence of calcium, the packed poloxamer 407 micelles allow the Ca^2+^ to diffuse, and a network gradually forms. This hypothesis helps explain the gradual erosion in the presence of calcium, but it still must be experimentally verified.

**Figure:**
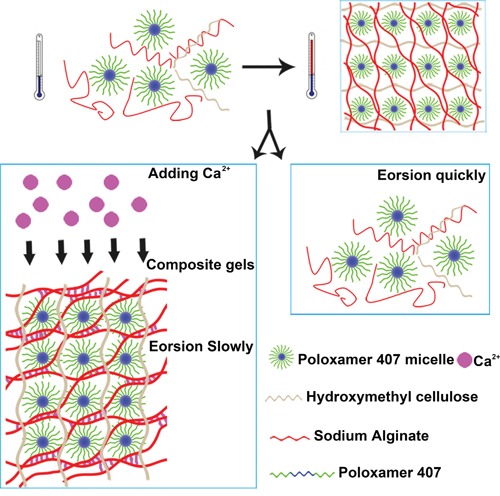
Scheme 1: Proposed schematic illustration for possible embolization mechanisms of PSHI-Ca^2+^ thermo-sensitive composite hydrogels.

P407, SA and HPMC are biocompatible polymers, and cytotoxicity was not expected from mixing these polymers. The results of this study demonstrated that PSHI had low cytotoxicity. However, conventional liquid embolic agents such as Onyx were toxic [27]. Overall, the composite hydrogel is suitable candidates for embolic delivery due to their low cytotoxicity.

The aim of this study was to demonstrate the ability of PSHI-Ca^2+^ to occlude a blood vessel *in vivo* and thus confirm its potential as a liquid embolic agent intransarterial embolization therapy. In this work, PSHI-Ca^2+^ was shown to successfully occlude rabbit normal renal artery and tumor. Moreover, the necrosis level and gel distribution indicated that the composite hydrogel had occluded the peripheral blood vessels during TAE therapy. In addition, an *ex vivo* histological analysis of the major organs after treatment showed no noticeable damage to the organs and a low toxicity of localized treatment. While, the percutaneous ethanol injection could damage the liver and cause serious painful to patients [28]. Thus, TAE therapy with thermo-sensitive composite PSHI-Ca2+ hydrogels could be a promising embolic agent for liver tumor treatment. PSHI-Ca^2+^ exhibited excellent embolism efficacy, the injection method was complicated. The injection method should be improved in future studies. The duration and stability of the embolization and the post-embolization drawback should be optimized. Further researches should be taken to explore the characteristics of drug loading PSHI-Ca^2+^ for chemoembolic-based tumor treatment.

## MATERIALS AND METHODS

### Materials

Poloxamer 407 (P407) and hydroxymethyl cellulose (HPMC-100M) were purchased from BASF Co., Ltd. (Shanghai, China). Sodium Alginate (SA), Calcium Chloride (CaCl_2_), 3-(4, 5-dimethyl-thiazol-2-yl)-2, 5-diphenyltetrazolium bromide (MTT) were obtained from Sigma-Aldrich Co., Ltd. (Shanghai, China). A nonionic agent Visipaque ^®^ 320 containing Iodixanol (320 mg I/ml) was purchased from GE Healthcare Inc. (Ireland Cork, Ireland). All solutions were prepared in Milli-Q Utrapure water (18.2 MΩ).

L929 cells were purchased from the Chinese Academy of Sciences (Shanghai, China), and the VX2 carcinoma strain was obtained from the American Type Culture Collection (ATCC). L929 cells were cultured in DMEM containing 10% fetal bovine serum supplemented with 1% penicillin and streptomycin in an incubator at 37°C in a 5% CO_2_ atmosphere. The VX2 tumor strain was maintained by successive transplantation into the hind legs of carrier rabbits.

New Zealand rabbits (Wt 2.0~2.5 kg, both sexes) were provided by the Laboratory Animal Research Center of the Shanghai Medical College of Fudan University (Shanghai, China). All experiments were conducted in accordance with the Animal Care Committee on the Guide for the Care and Use of Laboratory Animals.

### Preparation of the PSHI hydrogels

P407, sodium alginate, hydroxymethyl cellulose and Iodixanol composite hydrogels (P407-sodium alginate-HPMC-iodixanol hydrogel, PSHI) were prepared according to a previously reported cold method [29]. A sodium alginate (0.5% w/v) -HPMC(0.5% w/v) solution was made by dissolving alginate and hydroxymethyl cellulose powder in MilliQ water containing 23% (v/v) iodixanol with stirring overnight in the dark at room temperature. To prepare PSHI hydrogels with various P407 mass ratios, an appropriate volume of the sodium alginate-hydroxymethyl cellulose solution was transferred into a flat bottomed vial and placed in ice bath with stirring. Appropriate amounts of P407 were added to reach a final concentration of 17%, 18%, 19%, 20%, 21% and 22%.

### Sol-Gel transition characteristics

The thermo-sensitivity of the PSHI composite hydrogels was measured using the visual tube inversion method [30]. The sol-gel transition behavior was evaluated in this study. Briefly, 3 ml of PSHI was transferred into a 20-mm-diameter glass vial and incubated in a 37°C water bath. The time when the solution stopped flowing was reported as the gelation time. The PSHI composite hydrogels were also incubated at other temperatures (0°C, 4°C and 25°C) and subsequently gelled at 37°C. The gelation time for the various temperature differences was also recorded (n=3).

### Rheology of PSHI aqueous solution

To investigate the changes in the rheological characteristics of PSHI, the hydrogels were added to a lower stationary plate containing an inverted stainless steel plate. Rheology was evaluated by flow analysis and oscillation temperature sweeping (15°C-40°C) at predetermined time points with a rheometer (Malvern Kinexus Ultra, USA) in an oscillation mode of 1 Hz with a heating rate of 1°C/min. The storage modulus (G’), loss modulus (G“) and viscosity (η) of different concentrations of PSHI were measured by the change in heat (n=3).

### *In vitro* hydrogel erosion

*In vitro* erosion was evaluated using a membraneless model [31]. The hydrogel erosion characteristics were determined using a gravimetric method [32, 33]. PSHI solution (1 ml) was added to a vial and weighed. A composite hydrogel was subsequently formed by adding 0.5 ml of a calcium chloride solution to the preloaded PSHI gel. After mixing, the solution was removed from the composite hydrogel to examine the erosion behavior. Then, 1 ml PBS was added to the surface of the composite hydrogel and shaken for 30 min at 37°C. The fluid was removed, and the vial was reweighed. The weight loss was used to evaluate the erosion of the composite hydrogels using the following equation: weight loss (%) = [(W_i_-W_0_)/W_i_] × 100%, where W_i_ and W_0_ are the weight of the hydrogel at time i and time 0, respectively.

### Calcium-binding determination

The concentrations of calcium ion (Ca^2+^) were determined with a model of AAS vario z2000 atomic absorption spectrometer (Hitachi Japan) in flame mode.

### *In vitro* cytotoxicity study

An *in vitro* cytotoxicity test was performed on the L929 cells using PSHI-Ca^2+^ extracts at various concentrations (100%, 50%, 25%, 12.5% and 6.25%), as in previous studies [34, 35]. Extracts of PSHI-Ca^2+^ composite hydrogels were obtained according to a published method [36]. The extracts of the PSHI-Ca^2+^ composite hydrogels was diluted with culture medium. Cells were seeded in a 96-well plate (1×10^4^ cells/well) and cultured overnight, after which the medium was changed to the test sample extracts. After incubation for 24 h, 48 h and 72 h, the cell viability was assessed by the MTT assay.

### *In vivo* experiments

#### Renal arterial embolization of normal rabbits

After a rabbit was anesthetized with 1% sodium pentobarbital (30 mg/kg, intravenously) and fixed, the right femoral artery was dissected. A 4F arterial sheath was inserted into the femoral artery, lubricated with sodium heparin (100IU/kg) and positioned into the orifice of the renal artery, guided by fluoroscopy. A pre-embolization renal arteriogram was obtained by injecting 2 ml of contrast media. A 3F microcatheter was then placed into the 4F catheter through a hemostatic Y valve and advanced as close as possible to the main artery so the thermo-sensitive composite hydrogel (PSHI-Ca^2+^) could be injected. When the thermo-sensitive hydrogel began to reflux, the microcatheter was removed. Angiographic images were taken of the renal arteries before embolization and at 5 min and 30 min after embolization. After the radiography procedure, the femoral artery was bound, and the animals were returned to the laboratory [37].

### TAE treatmentof VX2 liver tumors

A VX2 liver tumor rabbit model was established as previously described [38]. All VX2 rabbits were fed for 18-24 days after a VX2 tumor was implanted and examined with computed tomography scanning. After CT scanning, further studies evaluated the embolic efficacy of the PSHI composite hydrogel in the VX2 liver tumors. The TAE treatment procedure was similar to renal arterial embolization. The PSHI composite hydrogel was slowly injected guided by digital subtraction angiography (DSA), and then CaCl_2_ was carefully administered. In the control group, 0.9% saline solution and PSHI hydrogel was injected instead of the PSHI-Ca^2+^ separately. All VX2 liver tumors of rabbits were fed normally after the TAE treatment. All interventional procedures were performed in a DSA unit conductor under strict aseptic conditions.

### Histopathological analysis

At the appropriate time, the rabbits were sacrificed. The heart, liver, spleen, lungs, kidneys and tumors were collected and fixed. The tissue sections were stained with hematoxylin/eosin (H&E) (original magnification×50, 100) for histological analysis to investigate organ damage. Toluidine blue-eosin staining set was performed to investigate the polymer distribution; the polymer stained blue, and the tissue stained red. Furthermore, a terminal nucleotide transferase-mediated nick and labeling (TUNEL) assay was carried out to observe the tumor apoptosis.

### Statistical analysis

Statistical analyses of all measurements were carried out using the SPSS 17.0 statistical software. All data were expressed as the mean ± standard deviation for each group, and Student's t-test was performed to compare the two groups. P<0.05 was considered statistically significant.

## SUPPLEMENTARY MATERIALS FIGURE


