# Supplementary material for: Thermo-sensitive composite hydrogels based on poloxamer 407 and alginate and their therapeutic effect in embolization in rabbit VX2 liver tumors
Source: Oncotarget. 2016 Sep 1;7(45):73280–91. doi: 10.18632/oncotarget.11789 (PMC5341979; doi:10.18632/oncotarget.11789)
Supplement: Supplementary file 1 [file oncotarget-07-73280-s001.pdf]

Thermo-sensitive composite hydrogels based on poloxamer 407 and alginate and their therapeutic effect in embolization in rabbit VX2 liver tumors

SUPPLEMENTARY FIGURE

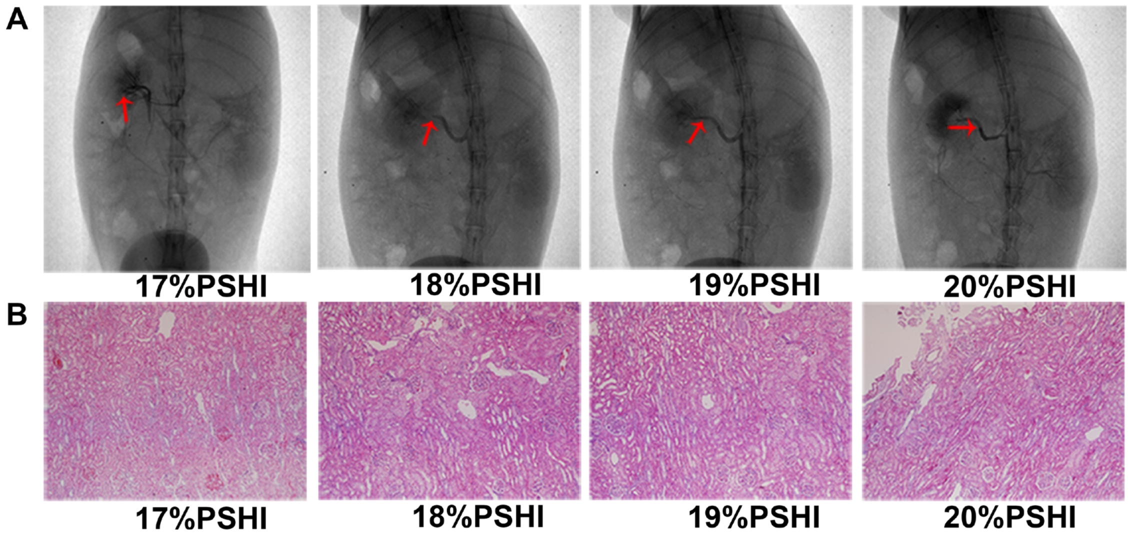

Supplementary Figure S1: Different P407 concentrations of PSHI in the renal arteries for embolization. A. DSA images of different formulations used for renal embolization. **B.** The histopathological analyses of rabbit kidney tissues after embolization. Supporting Information. Brief statement in nonsentence format listing the contents of the material supplied as Supporting Information.
